# Prevention of Adrenal Crisis: Cortisol Responses to Major Stress Compared to Stress Dose Hydrocortisone Delivery

**DOI:** 10.1210/clinem/dgaa133

**Published:** 2020-03-14

**Authors:** Alessandro Prete, Angela E Taylor, Irina Bancos, David J Smith, Mark A Foster, Sibylle Kohler, Violet Fazal-Sanderson, John Komninos, Donna M O’Neil, Dimitra A Vassiliadi, Christopher J Mowatt, Radu Mihai, Joanne L Fallowfield, Djillali Annane, Janet M Lord, Brian G Keevil, John A H Wass, Niki Karavitaki, Wiebke Arlt

**Affiliations:** 1 Institute of Metabolism and Systems Research, University of Birmingham, Birmingham, UK; 2 Centre for Endocrinology, Diabetes and Metabolism, Birmingham Health Partners, Birmingham, UK; 3 Division of Endocrinology, Metabolism and Nutrition, Department of Internal Medicine, Mayo Clinic, Rochester, MN; 4 School of Mathematics, University of Birmingham, Birmingham, UK; 5 Institute of Inflammation and Ageing, University of Birmingham, Birmingham, UK; 6 NIHR Surgical Reconstruction and Microbiology Research Centre, Queen Elizabeth Hospital, Birmingham, UK; 7 Royal Centre for Defence Medicine, Queen Elizabeth Hospital, Birmingham, UK; 8 Oxford Centre for Diabetes, Endocrinology and Metabolism, Churchill Hospital, Oxford, UK; 9 Department of Endocrinology, Diabetes and Metabolism, Evangelismos Hospital, Athens, Greece; 10 Department of Anaesthesiology, Royal Shrewsbury Hospital, The Shrewsbury and Telford Hospital NHS Trust, Shrewsbury, UK; 11 Department of Endocrine Surgery, Churchill Hospital, Oxford, UK; 12 Institute of Naval Medicine, Alverstoke, UK; 13 Critical Care Department, Hôpital Raymond-Poincaré, Laboratory of Infection & Inflammation U1173 INSERM/University Paris Saclay-UVSQ, Garches, France; 14 Department of Clinical Biochemistry, University Hospital of South Manchester, Manchester Academic Health Science Centre, The University of Manchester, Manchester, UK; 15 NIHR Birmingham Biomedical Research Centre, University of Birmingham and University Hospitals Birmingham NHS Foundation Trust, Birmingham, UK

**Keywords:** stress, surgery, hydrocortisone, cortisol, glucocorticoids, mass spectrometry

## Abstract

**Context:**

Patients with adrenal insufficiency require increased hydrocortisone cover during major stress to avoid a life-threatening adrenal crisis. However, current treatment recommendations are not evidence-based.

**Objective:**

To identify the most appropriate mode of hydrocortisone delivery in patients with adrenal insufficiency who are exposed to major stress.

**Design and Participants:**

Cross-sectional study: 122 unstressed healthy subjects and 288 subjects exposed to different stressors (major trauma [N = 83], sepsis [N = 100], and combat stress [N = 105]). Longitudinal study: 22 patients with preserved adrenal function undergoing elective surgery. Pharmacokinetic study: 10 patients with primary adrenal insufficiency undergoing administration of 200 mg hydrocortisone over 24 hours in 4 different delivery modes (continuous intravenous infusion; 6-hourly oral, intramuscular or intravenous bolus administration).

**Main Outcome Measure:**

We measured total serum cortisol and cortisone, free serum cortisol, and urinary glucocorticoid metabolite excretion by mass spectrometry. Linear pharmacokinetic modeling was used to determine the most appropriate mode and dose of hydrocortisone administration in patients with adrenal insufficiency exposed to major stress.

**Results:**

Serum cortisol was increased in all stress conditions, with the highest values observed in surgery and sepsis. Continuous intravenous hydrocortisone was the only administration mode persistently achieving median cortisol concentrations in the range observed during major stress. Linear pharmacokinetic modeling identified continuous intravenous infusion of 200 mg hydrocortisone over 24 hours, preceded by an initial bolus of 50–100 mg hydrocortisone, as best suited for maintaining cortisol concentrations in the required range.

**Conclusions:**

Continuous intravenous hydrocortisone infusion should be favored over intermittent bolus administration in the prevention and treatment of adrenal crisis during major stress.

The activation of the hypothalamic-pituitary-adrenal axis in response to stressful stimuli elicits increased glucocorticoid output aimed at restoring homeostasis. Cortisol is the major glucocorticoid produced by the human adrenal glands and is a key component of the physiological stress response (1).

Adrenal insufficiency is caused by failure of the adrenal cortex to produce cortisol, which can be caused by loss of function of the adrenal itself or its hypothalamic-pituitary regulatory center or, most commonly, long-term exogenous glucocorticoid treatment for other conditions. Patients with adrenal insufficiency are unable to produce adequate amounts of cortisol in response to stress and, therefore, require increased hydrocortisone replacement doses to avoid life-threatening adrenal crisis during surgery, trauma, or severe infection (2–4). Prevention of adrenal crisis is challenging (5, 6) and studies investigating the optimal dose and mode of steroid cover during major stress are lacking. Currently, administered hydrocortisone doses are chosen empirically rather than based on evidence. There is considerable variability in recommended administration modes, total doses, and dosing intervals (7). The lack of evidence-based recommendations for dose and mode of glucocorticoid replacement in major stress sends a confusing message to healthcare staff, which regularly exposes patients to harm (8).

This study was designed to determine the most appropriate hydrocortisone dose and delivery mode for patients with adrenal insufficiency during major stress. We employed tandem mass spectrometry to measure glucocorticoid concentrations in subjects with preserved adrenal function exposed to various conditions of stress and compared them to concentrations achieved after administration of stress dose hydrocortisone by a range of currently used delivery modes in patients with adrenal insufficiency.

## Materials and Methods

### Study design, participants, and procedures

**Figure 1.**
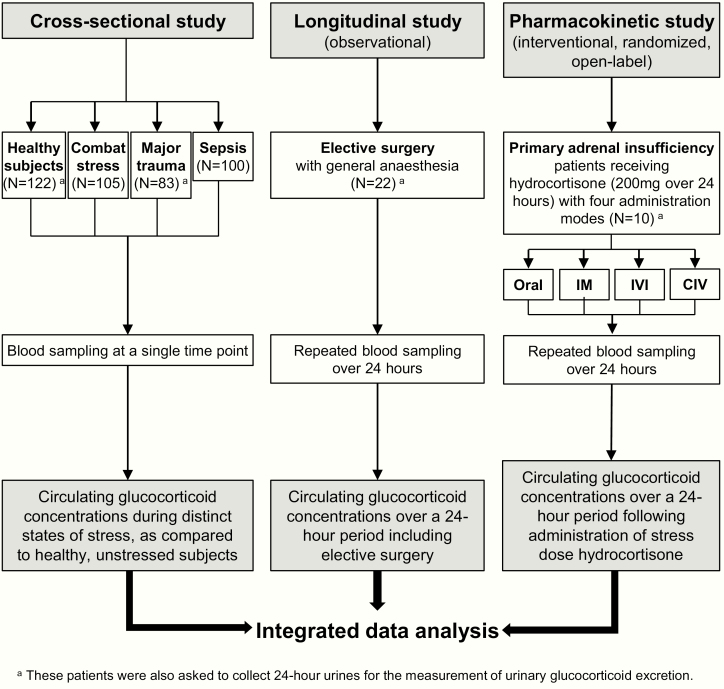
Summary of the studies performed. Assessment of the circulating and urinary glucocorticoid concentrations in response to different stress conditions and to stress dose hydrocortisone administration. Abbreviations: IM, intramuscular injection; IVI, intravenous injection; CIV, continuous intravenous infusion.

Three clinical studies were undertaken (Fig. 1), with patient demographics and outcome measures summarized in Table 1.

**Table 1.** Clinical characteristics of study participants and sampling regimen.

Cohort	Number of Subjects	Number of Females (%)	Age, Median (Range) Years	Time of Collection
Serum total cortisol/cortisone				
Healthy controls	122	58 (47.5%)	29 (20–69)	Between 9:00 and 11:00 am (single time point)
Subjects under combat stress	105	0	27 (19–47)	Between 6:00 and 9:00 am (single time point)
Patients with major trauma ^a^	83	9 (10.8%)	28 (18–85)	Within 24 hours of admission for major trauma (single time point)
Patients with sepsis	100	30 (30%)	71 (28–101)	Within 24 hours of fulfilling the criteria of sepsis (single time point)
Patients undergoing elective surgery ^b^	22	14 (63.6%)	49 (21–60)	24-hour profile from knife-to-skin onwards
Patients with primary adrenal insufficiency	10	8 (80%)	56 (40–64)	24-hour profile from 9:00 to 9:00 am
Serum free cortisol				
Patients with major trauma ^a^	18	4 (22.2%)	35 (19–75)	Within 3 days of admission (single time point)
Patients with sepsis	17	1 (5.9%)	63 (31–101)	Within 24 hours of fulfilling the criteria of sepsis (single time point)
Patients undergoing elective surgery ^b^	21	13 (61.9%)	49 (21–60)	At knife-to-skin and 4 hours after the initiation of surgery
Patients with primary adrenal insufficiency	10	8 (80%)	56 (40–64)	Two time points (T_min_ and T_max_) ^c^
24-hour urine glucocorticoid excretion				
Healthy controls	122	58 (47.5%)	29 (20–69)	During the day and night preceding the serum sample collection
Patients with major trauma ^a^	23	3 (13.0%)	41 (20–78)	Within 3 days of admission (24-hour collection)
Patients undergoing elective surgery ^b^	21	13 (61.9%)	49 (21–60)	24-hour collection from knife-to-skin onwards
Patients with primary adrenal insufficiency	10	8 (80%)	56 (40–64)	24-hour collection from 9:00 to 9:00 am

^a^All patients underwent measurements of serum total cortisol and cortisone. A subgroup of patients provided samples to measure serum free cortisol and urinary glucocorticoids.

^b^All patients underwent measurements of serum total cortisol and cortisone. All but 1 patient provided samples to measure serum free cortisol and urinary glucocorticoids.

^c^Blood was collected at T_min_: time when the minimum serum total cortisol levels were observed after hydrocortisone administration; and T_max_: time when the maximum serum total cortisol levels were observed after hydrocortisone administration.

First, in a cross-sectional study, we measured circulating glucocorticoid concentrations in 122 healthy, nonstressed controls and 288 subjects with distinct and defined states of stress at the time of blood sampling. These conditions of stress included: 105 otherwise healthy subjects under combat stress (blood samples taken within 4 weeks of their deployment to the Afghanistan conflict) (9); 83 prospectively recruited subjects with acute major trauma (estimated new injury severity score [NISS] > 15 (10); blood samples taken within 24 hours of acute injury, excluding brain injury); and 100 consecutively recruited patients with sepsis (blood samples collected within 24 hours of fulfilling the criteria for sepsis (11) in the intensive care unit setting). At the time of sampling, none of the subjects had an established diagnosis of adrenal insufficiency or were receiving treatment with glucocorticoids or other medications with a major impact on steroid synthesis or metabolism.

Second, we prospectively recruited 22 patients with normal adrenal function who underwent repeated longitudinal serum sample collection over a 24-hour period whilst undergoing elective surgery with general anesthesia (Supplementary Table 1) (12). Blood samples were drawn at the following time points: 0 (= knife-to-skin, KTS), 0.5, 1, 2, 3, 4, 5, 6, 12, and 24 hours.

Third, we undertook a randomized, open-label study in 10 patients with an established diagnosis of primary adrenal insufficiency and on stable steroid replacement therapy for at least 6 months (Supplementary Table 2) (12). All patients attended the clinical research facility for a 24-hour study period on 4 occasions separated by at least 1 week. On each study day, they were admitted at 8:00 am after an overnight fast and last intake of their regular steroid replacement at 12:00 pm the preceding day; standardized meals were served at 10:00 am, 2:00 pm, and 6:00 pm. On each of the study days, subjects received 200 mg hydrocortisone over 24 hours administered by 1 of 4 different administration modes: oral tablets (ORAL; 50 mg at 9:00 am, 3:00 pm, 9:00 pm, and 3:00 am); intramuscular bolus injection (IM; 50 mg at 9:00 am, 3:00 pm, 9:00 pm, and 3:00 am); intravenous bolus injection (IVI; 50 mg at 9:00 am, 3:00 pm, 9:00 pm, and 3:00 am); continuous intravenous infusion (CIV) of 200 mg hydrocortisone over 24 hours (diluted in 50 ml glucose 5% and administered via perfusor at a rate of 4 ml/hour). The 4 different administration modes were administered to each patient in random order (Supplementary Table 2) (12). Blood sampling was carried out every 30 minutes from 9:00–11:00 am, 3:00–5:00 pm, 9:00–11:00 pm, and 3:00–5:00 am, and otherwise in hourly intervals throughout the 24-hour study period.

### Ethics approval

All study participants provided written informed consent prior to inclusion and all study procedures underwent ethics committee approval prior to recruitment (combat stress: MOD REC 116/Gen/10; major trauma: NRES Committee South West—Frenchay 11/SW/0177; sepsis: Comité de Protection des Personnes de Saint-Germain-en-Laye—COITTSS trial NCT00320099; elective surgery and adrenal insufficiency: South Birmingham REC Ref 07/H1207/22).

### Glucocorticoid measurements

Serum concentrations of total cortisol and its inactive metabolite cortisone were measured by liquid chromatography-tandem mass spectrometry (LC-MS/MS) as previously described (13). For measurement of serum free cortisol concentrations, the unbound cortisol fraction was separated by temperature-controlled ultrafiltration, centrifuged in preconditioned ultrafiltration devices and then measured with LC-MS/MS, as previously described (14). Measurement of 24-hour urinary glucocorticoid excretion was carried out by gas chromatography/mass spectrometry, as previously described (15). For further details on the mass spectrometry analysis, see Supplemental Methods (12).

### Statistical analysis

Medians with 5^th^ to 95^th^ percentile ranges and interquartile ranges were calculated for continuous variables. The area under the concentration-time curve (area under a curve [AUC]) was calculated by means of trapezoidal integration. Serum cortisol concentrations between the various groups were compared by Kruskal–Wallis and Mann–Whitney U tests. The level of significance was set at *P* < 0.05. Statistical analyses were performed by SPSS 178 21.0 for Windows (SPSS, Inc., Chicago, IL) and MATLAB (Mathworks, Natick, MA).

### Pharmacokinetic modeling analysis

The serum cortisol time course response c(t) was modeled relative to intravenous hydrocortisone via linear pharmacokinetics, $\frac{dc}{dt}=-kc+q,\;c\left(0\right)=Q,$ where k is clearance rate, Q is initial response (representing intravenous bolus [IVI] delivery), and q is the rate of continuous intravenous (CIV) delivery of hydrocortisone. This model has the exact solution, $c\left(t\right)=Qe^{-kt}+\frac{q}{k}\left(1-e^{-kt}\right).$ Intravenous bolus 50 mg data over 6–12 hours was used to fit the parameters k and Q (with q=0) using a mixed-effects model implemented in MATLAB (Mathworks, Natick, MA) and the function *nlmefit*. This approach enabled the estimation of population average (fixed effects) and between-patient heterogeneity (random effects). Responses to other modes of administration were predicted by plotting model solutions with appropriately modified parameters *q* and *Q*; for example IVI 100 mg was modeled by taking *Q* = 2*Q* and *q* = 0; CIV 200 mg per 24 hours was modeled by taking q=Q/6 and Q=0.

## Results

### Glucocorticoid concentrations in different conditions of stress

**Figure 2.**
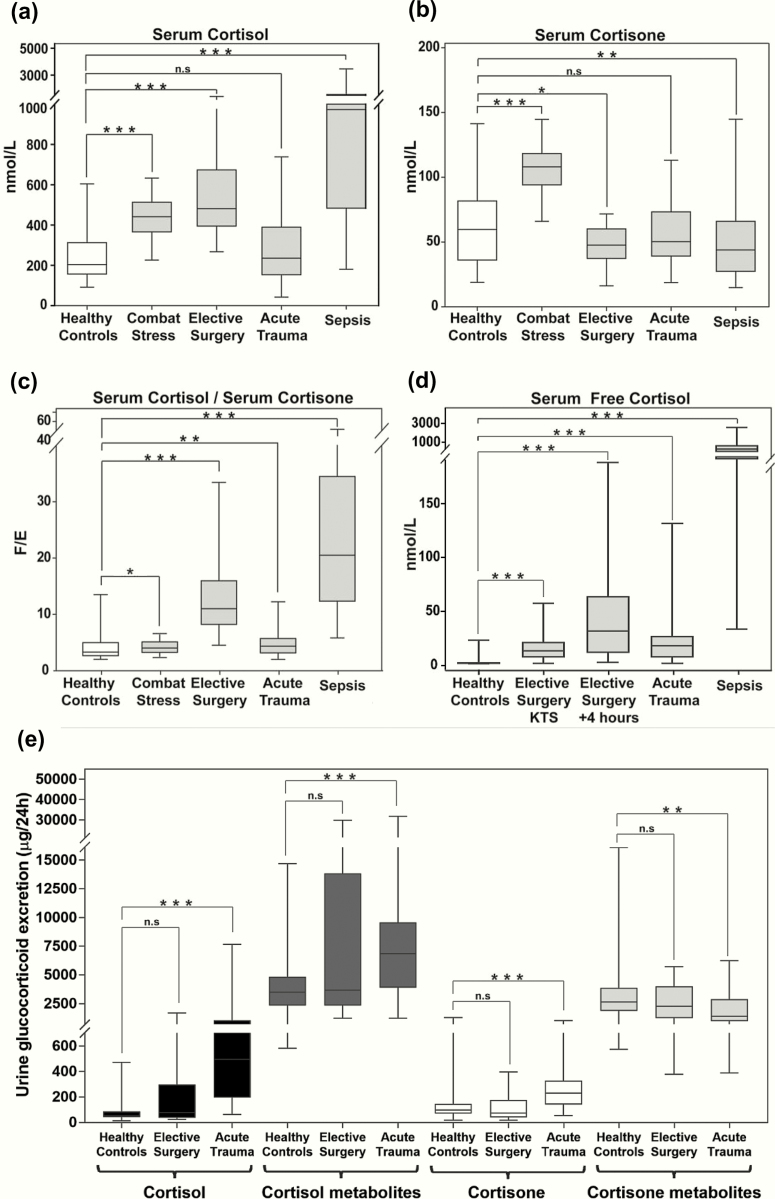
Circulating glucocorticoids during major stress. Serum concentrations of total cortisol (nmol/L) (**a**), total cortisone (nmol/L) (**b**), and cortisol (F)/cortisone (E) ratio (**c**) in healthy controls (N = 122), during combat stress (N = 105), during elective surgery (N = 22), after major trauma (N = 83), and during sepsis (N = 100). In the patients undergoing elective surgery, the maximum serum cortisol levels (and corresponding serum cortisone levels) were used for the calculations. Panel **d:** reports serum free cortisol concentrations in nmol/L in healthy controls (N = 11), during elective surgery at knife-to-skin (KTS, N = 21), and 4 hours after the start of the operation (N = 21), after major trauma (N = 18), and during sepsis (N = 17). Panel **e:** reports the 24-hour urinary excretion of cortisol, cortisol metabolites, cortisone, and cortisone metabolites in healthy controls (N = 122), following elective surgery (N = 21) and after major trauma (N = 23). Boxes show median and interquartile range, whiskers are 5^th^ to 95^th^ percentile. Symbols: n.s., *P > 0*.05; *, *P ≤ 0*.05; **, *P ≤ 0*.01; ***, *P ≤ 0*.001.

Serum total cortisol concentrations were highest and most variable in patients with sepsis, followed by patients undergoing elective surgery with general anesthesia, patients with combat stress, and patients with acute major trauma (Fig. 2A). Pairwise comparisons showed significant differences between unstressed controls versus all stressed groups, except for patients with major trauma.

When analyzing the inactive cortisol metabolite cortisone, pairwise comparisons to levels observed in unstressed controls showed significantly higher serum cortisone in combat stress, while circulating cortisone was significantly lower in elective surgery and sepsis patients; serum cortisone concentrations in patients after major trauma did not differ from unstressed controls (Fig. 2B). The serum cortisol/cortisone ratio showed a significant increase, favoring active cortisol in all stress conditions, with the highest increase in sepsis (Fig. 2C).

Free serum cortisol concentrations were higher than in unstressed controls in all stressed groups, with the highest concentrations observed in sepsis (Fig. 2D).

Twenty-four-hour urinary excretion of cortisol, cortisone, and their major metabolites was significantly increased in major trauma, while glucocorticoid excretion in patients undergoing elective surgery did not significantly differ from unstressed controls (Fig. 2E).

### Glucocorticoid dynamics during elective surgery

We analyzed—separately—the circulating glucocorticoid concentrations in patients undergoing surgeries of a short duration (median duration 60 minutes, range 25–85 minutes; n = 11) from those who underwent a longer-lasting surgery (median duration 175 minutes, range 100–295 minutes; n = 11). In both groups, serum cortisol decreased within an hour of induction of anesthesia, followed by a gradual increase. In the group with a shorter surgery, maximum serum cortisol concentrations (C_max_) were observed after a median of 3 hours post-KTS, while in the group with a surgery of a longer duration, C_max_ were observed after a median of 5 hours post-KTS (Table 2 and Supplementary Fig. 1) (12), ie, during the wake-up phase after general anesthesia.

**Table 2.** Pharmacokinetic parameters of serum total cortisol concentrations observed during elective surgery in patients with preserved adrenal function (N = 22) and after hydrocortisone administration via four different modes in patients with primary adrenal insufficiency (N = 10)

**Elective Surgery**	**0–24 h**						**0–6 h**	**6–12 h**	**12–24 h**	
	**C**_**max**_ **(nmol/L)**	**T**_**max**_ **(h)**	**C**_**min**_ **(nmol/L)**	**T**_**min**_ **(h)**	**ΔC**_**max**_ **-C**_**min**_	AUC (nmol*h/L)	AUC (nmol*h/L)	AUC (nmol*h/L)	AUC (nmol*h/L)	
Patients with normal baseline adrenal function undergoing elective surgery with general anesthesia										
All patients (N = 22)	522 (261–1379)	4 (0–12)	60 (17–320)	2 (0–24)	423 (220–1287)	5295 (1191–22 274)	1812 (285–5687)	1329 (324–7221)	2154 (582–9366)	
Surgery of longer duration (N = 11)	611 (261–1379)	5 (0–12)	66 (17–320)	2 (0–24)	499 (235–1287)	8026 (1343–22 061)	2107 (338–5474)	2433 (393–7221)	3486 (612–9366)	
Surgery of shorter duration (N = 11)	431 (261–1379)	3 (0–12)	56 (17–320)	1 (0–24)	375 (235–1287)	3922 (1492–10 807)	1681 (502–3517)	807 (324–2718)	1434 (666–4572)	
**Primary adrenal insufficiency**	**0–24 h**						**0–6 h**	**6–12 h**	**12–18 h**	**18–24 h**
	**C**_**max**_ **(nmol/L)**	**T**_**max**_ **(h)**	**C**_**min**_ **(nmol/L)**	**T**_**min**_ **(h)**	**ΔC**_**max**_ **-C**_**min**_	AUC (nmol*h/L)	AUC (nmol*h/L)	AUC (nmol*h/L)	AUC (nmol*h/L)	AUC (nmol*h/L)
Patients with primary adrenal insufficiency (N = 10) receiving 200 mg hydrocortisone over 24 hours in 4 different delivery modes										
ORAL (50 mg/6 h)	1423 (1083–2457)	0.5 (0.5–1.5)	277 (64–398)	6 (0.5–6)	1089 (834–2393)	15 267 (8591–22 417)	3807 (2471–5731)	4056 (1839–6348)	4200 (2539–5700)	3944 (2208–5600)
IM (50 mg/6 h)	1152 (830–1345)	1 (0.5–2)	289 (148–453)	6 (6)	844 (581–1151)	14 950 (10 383–20 102)	3887 (2864–5200)	4055 (2429–5296)	3781 (2789–5135)	3866 (2711–5305)
IVI (50 mg/6 h)	1449 (1072–2432)	0.5 (0.5–6)	171 (0–375)	6 (6)	1239 (954–2261)	13 413 (9412–20 220)	3577 (2415–4852)	3466 (2623–4815)	3453 (2440–6084)	3425 (2310–5253)
CIV (200 mg/24 h)	836 (661–1073)	7 (2–18)	520 (388–617)	20 (12.5–23.0)	329 (232–551)	14 649 (10 934–19 082)	3582 (2685–5025)	4067 (2938–5112)	4004 (3033–5069)	3712 (2796–4766)

The table reports pharmacokinetic parameters determined from circulating serum total cortisol concentrations observed in 22 patients undergoing elective surgery with general anesthesia (knife-to-skin = 0 hour) and after the administration of 200 mg hydrocortisone over 24 hours to 10 patients with primary adrenal insufficiency. Hydrocortisone was administered either as 6-hourly bolus injection (ORAL, IM, IVI) or by CIV. All data are presented as median (range); numbers for the 3 different hydrocortisone bolus administration modes represent averages of the observations made during the 4 consecutive 6-hour intervals, while CIV data refer to the time period 2–24 hour (steady state was achieved at 2 hours during CIV).

Abbreviations: CIV, continuous intravenous infusion; C_max_, maximum serum total cortisol concentration observed; C_min_, minimum serum total cortisol concentration observed; IM, intramuscular; IVI, intravenous injection; T_max_, time when the maximum serum total cortisol concentrations (C_max_) were observed; T_min_, time when the minimum serum total cortisol concentrations (C_min_) were observed.

After reaching C_max_, both serum cortisol and cortisone concentrations gradually decreased back to the presurgical baseline levels in the patients with a short duration surgery, while circulating glucocorticoid concentrations remained increased in the group with longer-lasting surgery (Supplementary Fig. 1) (12). The serum cortisol/cortisone ratio followed a similar pattern, with no difference between the 2 groups after 24 hours (Supplementary Fig. 1) (12).

### Pharmacokinetics of stress dose hydrocortisone in patients with primary adrenal insufficiency

**Figure 3.**
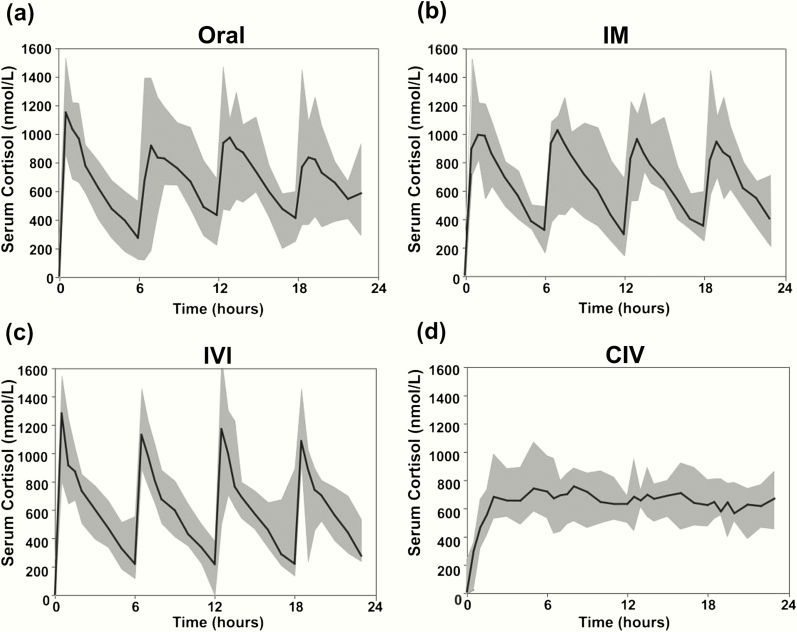
Serum total cortisol following hydrocortisone administration. Serum total cortisol (nmol/L) in 10 patients with adrenal insufficiency after hydrocortisone administered ORAL, IM, as IVI, and as CIV. Data are presented as median (black line) and range (shaded grey area).

After the administration of bolus hydrocortisone, C_max_ were achieved after a median time of 30 minutes (ORAL and IVI) or 60 minutes (IM), followed by a decrease to minimum concentrations (C_min_) after a median time of 360 minutes, ie, before the administration of the next 6-hourly dose (Fig. 3A–3C and Table 2). By contrast, CIV administration of hydrocortisone led to serum cortisol concentrations persistently within the same range from around 2 hours after the commencement of infusion, without distinct peak and trough concentrations after the achievement of steady state (Fig. 3D). Serum cortisone concentrations remained stable throughout, with no notable differences between the 4 hydrocortisone delivery modes (Supplementary Fig. 2) (12).

For all 4 hydrocortisone administration regimens, serum free cortisol concentrations at T_max_ (ie, when C_max_ were observed) were significantly higher than those observed in patients exposed to different stress conditions, except for sepsis, where free cortisol tended to be higher (Supplementary Fig. 3A) (12). Free cortisol during CIV at T_min_ (ie, when C_min_ were observed) was significantly higher than in surgical patients at KTS and 4 hours into surgery, and after acute trauma, but significantly lower than in sepsis (Supplementary Fig. 3A) (12). Free cortisol concentrations at T_min_ of the other hydrocortisone administration protocols were significantly lower than in sepsis but did not differ from those observed during other stress conditions.

The pattern of 24-hour urinary glucocorticoid metabolite excretion was similar in patients receiving hydrocortisone in the IM, IV, and CIV administration modes while after oral hydrocortisone administration, urine cortisol excretion was lower but cortisol metabolite excretion was higher (Supplementary Fig. 3B) (12), indicative of a first-pass effect with rapid metabolism of cortisol to downstream tetrahydro-metabolites in the liver. Glucocorticoid metabolite excretion after exogenous hydrocortisone administration resembled the pattern observed in major trauma, while patients with elective surgery and unstressed controls had a much higher proportion of cortisone metabolites (Supplementary Fig. 3B) (12).

### Serum cortisol after hydrocortisone administration versus serum cortisol during elective surgery

**Figure 4.**
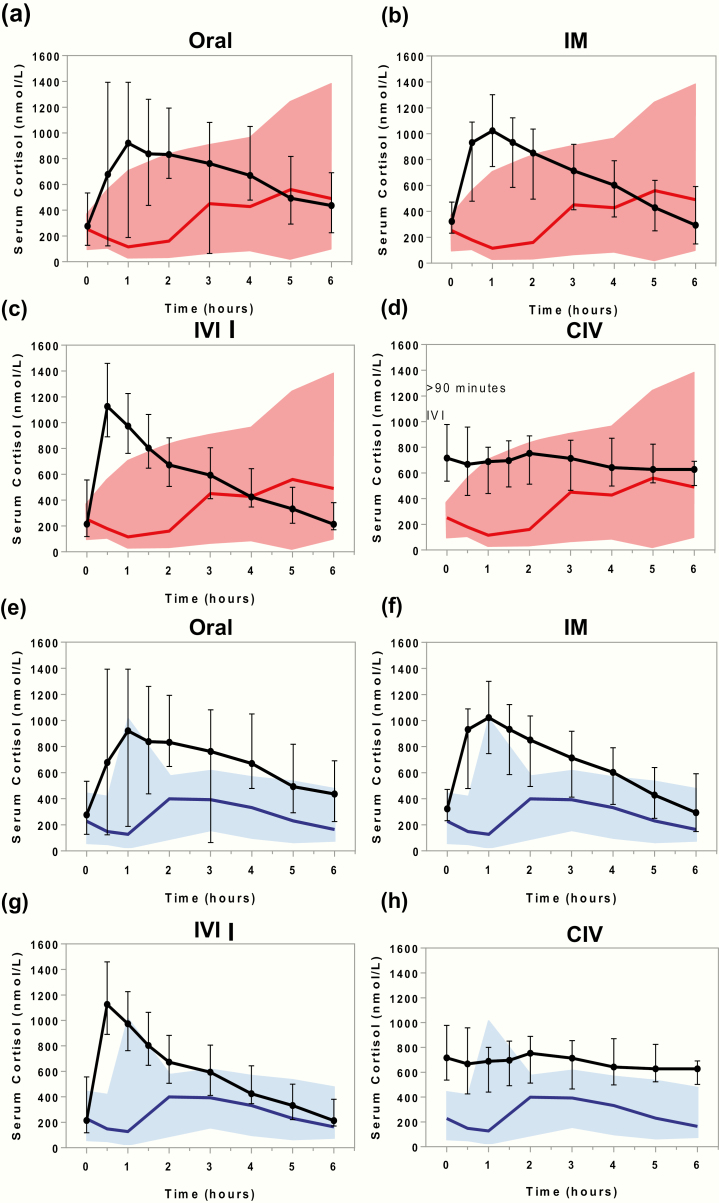
Comparison of serum total cortisol during elective surgery of longer duration and following hydrocortisone administration. Serum total cortisol concentrations (nmol/L) in 10 patients with adrenal insufficiency after the administration of 50 mg hydrocortisone over 6 hours (black line: median, whiskers: range) in 4 different modes (ORAL, IM or IVI, or as CIV) projected onto serum cortisol concentrations observed in patients undergoing elective surgery (panels **a**–**d**, serum cortisol in 11 patients undergoing elective surgery of longer duration [red line: median; red shaded area: range]; panels **e**–**h**, serum cortisol in 11 patients undergoing elective surgery of shorter duration [blue line: median; blue shaded area: range]) from time point knife-to-skin (KTS; 0 hours) to 6 hours post-KTS. All measurements were carried out by tandem mass spectrometry.

Serum cortisol concentrations observed in the 10 patients with primary adrenal insufficiency after hydrocortisone administration were plotted against the cortisol response of patients undergoing surgery of longer (N = 11; Fig. 4A–4D) and shorter duration (N = 11; Fig. 4E–4H). Initial peak cortisol concentrations after hydrocortisone administration in the primary adrenal insufficiency patients exceeded the concentrations observed during elective surgery in patients with preserved adrenal function. However, median cortisol concentrations after ORAL, IM, and IVI hydrocortisone administration decreased to trough levels below the median observed in patients undergoing longer-lasting surgery several hours before the scheduled repeat administration of bolus hydrocortisone (Fig. 4A–4C). By contrast, CIV hydrocortisone administration persistently maintained serum cortisol concentrations above the median of concentrations observed in patients undergoing elective surgery (Fig. 4D).

Serum cortisone concentrations in primary adrenal insufficiency and surgical patients showed a similar pattern; again, only CIV hydrocortisone administration achieved concentrations consistently above those observed in subjects undergoing elective surgery (Supplementary Fig. 4) (12).

### Linear pharmacokinetic modeling of stress dose hydrocortisone administration

Next, we used the pharmacokinetic data obtained in the primary adrenal insufficiency patients undergoing exogenous hydrocortisone administration to model the most appropriate dose and mode of hydrocortisone delivery for raising cortisol concentrations quickly and sustain concentrations within the desired range, defined as above the median observed during elective longer-lasting surgery. Fitting to IVI, serum total cortisol concentrations yielded parameter estimates for the fixed effect (average) of initial response *Q* = 1347 nmol/L (SE 70nmol/L) and clearance rate *k* = 0.27 h^-1^ (SE 0.016 h^-1^). Random effect variances were calculated as (158 nmol/L)^2^ and approximately 0, respectively.

**Figure 5.**
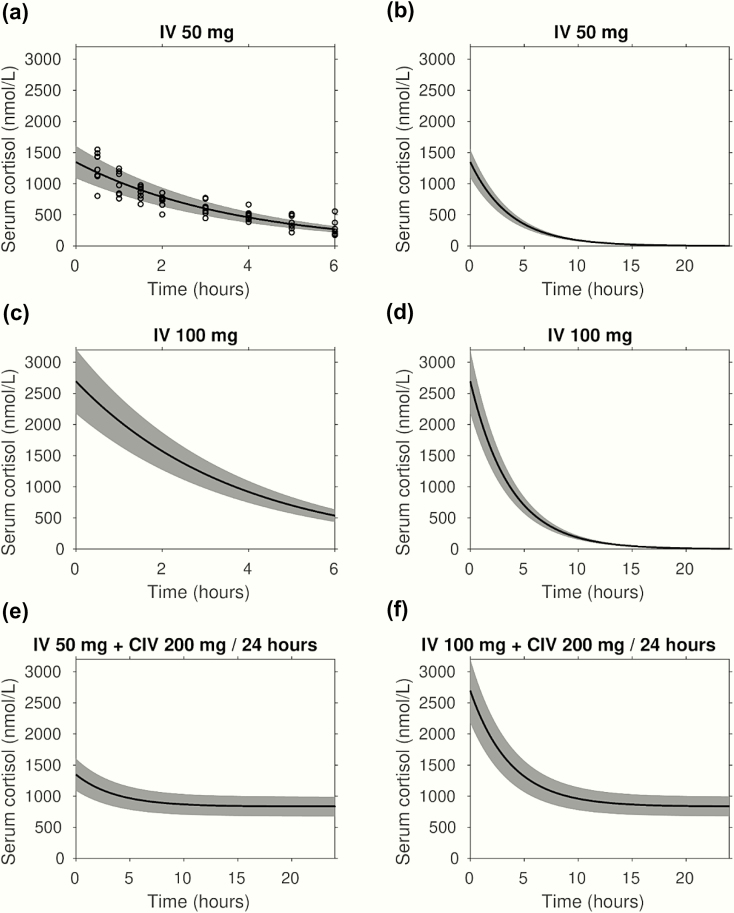
Linear pharmacokinetic modeling of stress dose hydrocortisone administration. Mixed effects linear pharmacokinetic modeling of serum cortisol in response to intravenous hydrocortisone administration modes. Serum cortisol concentrations are presented in nmol/L; the black lines show the fixed effect (central tendency) kinetics, the shaded gray area indicates 90% of between-patient variability. Panels **a** and **b**: show cortisol measurements (circles) following 50 mg IV bolus injection and the fitted model over 6 and 24 hours, respectively. The pharmacokinetic modeling was also used to predict the serum cortisol response to 100 mg IV bolus injection over 6 and 24 hours (panels **c** and **d**, respectively), as well as initial 50 mg (panel **e**) and 100 mg (panel **f**) IV bolus injections followed by CIV infusion of 200 mg per 24-hour hydrocortisone.

Fig. 5A depicts the 5^th^ and 95^th^ percentile range modeled on the serum cortisol concentrations observed after IV bolus injection of 50 mg hydrocortisone dose; Fig. 5B shows the predicted 24-hour serum cortisol concentrations. The model and fitted parameters were used to predict the serum cortisol responses to 3 alternative modes: 100 mg hydrocortisone IV bolus injection (Fig. 5C and 5D) and initial 50 mg (Fig. 5E) and initial 100 mg (Fig. 5F) IV bolus injections, both followed by CIV infusion of 200 mg per 24-hour hydrocortisone. Modeling of these 2 regimens predicted that both would achieve the serum cortisol concentration range observed for longer-lasting elective surgery, with a near-instantaneous initial increase in serum cortisol concentration.

## Discussion

Patients with adrenal insufficiency are unable to mount a cortisol response to counteract a stressful event and, therefore, their regular replacement dose needs to be increased during major stress to avoid adrenal crisis (16). Nevertheless, no consensus exists regarding the optimal dose and hydrocortisone delivery mode during major stress, and current recommendations are empirical rather than evidence-based (7, 17). This study is the first systematic dose-response study comparing the cortisol dynamics after the administration of stress doses of hydrocortisone in patients with adrenal insufficiency to the acute cortisol response induced by surgery and other conditions of major stress. Our aim was to define the most clinically appropriate but still practically feasible regimen of hydrocortisone administration during major stress in patients with adrenal insufficiency based on state-of-the-art tandem mass spectrometry measurements of circulating glucocorticoids. We found that continuous intravenous hydrocortisone was the only delivery mode that steadily maintained circulating cortisol in the range observed during major stress, while intermittet bolus administration of hydrocortisone resulted in frequent troughs with lower concentrations, thereby potentially exposing patients with adrenal insufficiency to periods of under-replacement, and hence the possibility of adrenal crisis, a life-threatening complication of cortisol deficiency.

In line with previously reported findings (18–20), we documented that serum total cortisol concentrations in all examined conditions of psychological and physical stress were increased above those observed in healthy, unstressed controls. The only exception was major trauma, with relatively lower total serum cortisol concentrations but increased serum free cortisol and 24-hour urinary cortisol, likely explained by the impact of blood loss in these patients.

Consistent with our previous systematic review and meta-analysis of the cortisol response to surgery (20), we observed an initial decrease in serum cortisol during elective surgery, which is likely to be linked to the induction of anesthesia. We observed higher cortisol concentrations during longer-lasting surgeries, using reference standard tandem mass spectrometry for serum glucocorticoid analysis. This was also observed in a recent study in 93 patients undergoing elective surgery (21), with serum cortisol measurements carried out by immunoassay. In a previous meta-analysis of studies investigating the serum cortisol response to surgery (20) we did not find an impact of the duration of surgery on peri- and postoperative serum cortisol concentrations, likely explained by the heterogeneity of the studies included, which were also limited by the near exclusive use of immunoassays and lack of measurement of free cortisol.

Linear pharmacokinetic modeling of stress dose hydrocortisone administration modes and doses combined with mixed effects regression identified continuous intravenous infusion of 200 mg hydrocortisone over 24 hours as the most appropriate replacement regiment in patients with adrenal insufficiency exposed to major stress. Modeling indicated that this should be preceded by a one-off initial intravenous bolus of 50–100 mg hydrocortisone to rapidly increase serum cortisol and shorten the time to steady state. We found that continuous intravenous hydrocortisone infusion was the only delivery mode to maintain cortisol concentrations persistently in the range observed during major stress, including longer-lasting surgery. This regimen did not result in significant peaks and troughs in circulating cortisol, which were observed with the 3 hydrocortisone bolus administration modes (ORAL, IM, and IVI). Significant troughs potentially expose patients with adrenal insufficiency to under-replacement and the risk of life-threatening adrenal crisis, while supraphysiologic peaks might come with adverse side effects, as previously shown in the context of sepsis with an increased rate of hyperglycemic episodes (22).

A major strength of the present study is the use of reference standard tandem mass spectrometry for the measurement of circulating glucocorticoid concentrations, with all samples measured contemporaneously and with the same assay. Traditional immunoassays are associated with considerable interassay variation and potential cross-reactivity with other steroids, which may lead to over- and underestimations of true levels in critically ill patients with stress-induced stimulation of the hypothalamic-pituitary-adrenal axis (23, 24). We also used mass spectrometry for the direct measurement of serum free cortisol, an important strength in comparison to studies who only employed indirect calculation of serum free cortisol utilizing cortisol-binding globulin, which is often inaccurate in the context of acute surgery and critical illness (20, 25, 26). Our previous systematic review and meta-analysis (20) only identified 2 studies measuring perioperative glucocorticoids by tandem mass spectrometry (27, 28) and 2 studies directly measuring serum free cortisol in patients undergoing surgery (28, 29).

One of the limitations of the present study is that while serum cortisol was measured during elective surgery repeatedly over a 24-hour period, concentrations for the other stress conditions were measured at a single time point only. In the acute phase, sepsis causes a surge of circulating cortisol to persistently raised concentrations (18). Though we observed the highest serum cortisol concentrations in sepsis, it is unlikely that hydrocortisone doses higher than 200 mg per 24 hours would be required to cover patients with adrenal insufficiency in that situation, as critical illness results in a decrease in cortisol inactivation (30). Moreover, in the context of patients with sepsis but normal adrenal function prior to illness, an increase from hydrocortisone 200 mg per 24 hours to 300 mg per 24 hours did not impact morbidity or mortality (31). In the present study, we did not assess the dynamics of cortisol metabolism during surgery and major stress. The previously reported reduced cortisol clearance during critical illness (30) may affect the requirements of hydrocortisone in patients with adrenal insufficiency and should be taken into consideration when interpreting our findings. A recent study reported that 100 mg hydrocortisone per 24 hours might be sufficient, though this was based on data collected in mostly secondary AI patients with likely residual cortisol biosynthetic capacity, with measurements carried out by immunoassays (32). Another limitation of our study is that the surgical group comprised mostly patients undergoing moderately invasive procedures. Thus, we cannot exclude that more invasive and longer-lasting surgeries could yield even higher serum cortisol concentrations. However, maximum cortisol concentrations were usually observed after the end of surgery in our patients, likely coinciding with the withdrawal of general anesthesia, although more invasive surgeries will be undertaken with the appropriately anesthesia and pain control regimens, thus not necessarily eliciting a higher cortisol response. We could not analyze the differential effects of the 4 different hydrocortisone regimens on mineralocorticoid activity in the context of our study; however, as 50 mg hydrocortisone are equivalent to 250 μg fludrocortisone (33), it is safe to assume that all 4 administration modes of 200 mg hydrocortisone per 24 hours will deliver more than sufficient mineralocorticoid activity.

In conclusion, our data provide evidence that hydrocortisone stress dose cover during surgery, trauma, and major illness in patients with adrenal insufficiency should be provided by continuous intravenous infusion of 200 mg hydrocortisone over 24 hours, following the administration of an initial intravenous hydrocortisone bolus of 50–100 mg.

The required duration of such stress dose cover is an important consideration and data on circulating cortisol concentrations beyond 2 days after the onset of major physical stress are scarce (20). However, a recently published study has followed patients with major trauma from injury to 6 months after recovery, describing increased urinary cortisol metabolite excretion for up to 8 weeks after trauma and increased cortisol reactivation by 11β-hydroxysteroid dehydrogenase type 1 peaking at 2 weeks after severe injury and normalizing by 8 weeks (34). In essence, the ability to taper back to normal replacement doses will depend on whether significant systemic inflammation is still present, if the patient is still looked after in the intensive care unit setting, and is nil by mouth. With regard to elective surgery, the Endocrine Society’s US primary adrenal insufficiency guidelines (17) and the recent UK guidelines for the perioperative management of glucocorticoid replacement in adrenal insufficiency (35) recommend that high-dose glucocorticoid replacement should be tapered back to the routine maintenance dose within 48 hours, extending this to up to a week if surgery is more major or complicated, with clinical judgement used to guide this process. Both guideline groups had access to the results of this study, which in both instances resulted in expert consensus to recommend continuous intravenous infusion as the preferred administration mode for hydrocortisone during major stress (17, 35).

## Additional Information

***Disclosure Summary:*** The authors have nothing to disclose.

***Data Availability:*** The datasets generated during and/or analyzed during the current study are not publicly available but are available from the corresponding author on reasonable request.
